# Short sequence motif dynamics in the SARS-CoV-2 genome suggest a role for cytosine deamination in CpG reduction

**DOI:** 10.1093/jmcb/mjab011

**Published:** 2021-02-25

**Authors:** Mukhtar Sadykov, Tobias Mourier, Qingtian Guan, Arnab Pain

**Affiliations:** 1 King Abdullah University of Science and Technology (KAUST), Pathogen Genomics Laboratory, Biological and Environmental Science and Engineering (BESE), Thuwal-Jeddah 23955-6900, Saudi Arabia; 2 Research Center for Zoonosis Control, Global Institution for Collaborative Research and Education (GI-CoRE), Hokkaido University, N20 W10 Kita-Ku, Sapporo 001-0020, Japan

**Keywords:** virus evolution, genome evolution, genome biology, virus‒host interaction

**Dear Editor,**

The apolipoprotein B editing complex (APOBEC) protein family members are host antiviral enzymes known for catalyzing cytosine to uracil (C>U) deamination in foreign single-stranded DNA (ssDNA) and RNA (ssRNA) (Blanc and Davidson, 2010; Salter and Smith, 2018). Enzymatic target motifs for most of the APOBEC enzymes have been experimentally identified, among which the most common ones are 5′-[T/U]C-3′ and 5′-CC-3′ for DNA/RNA substrates (Salter and Smith, 2018; McDaniel et al., 2020). It was recently suggested that SARS-CoV-2 undergoes genome editing by host-dependent RNA-editing proteins such as APOBEC (Di Giorgio et al., 2020; Rice et al., 2020; Simmonds, 2020; Schmidt et al., 2021).

Given the large amount of available data and the relatively low mutation rate of the SARS-CoV-2 virus (Rambaut et al., 2020), we aimed to monitor its genomic evolution on a very brief time scale during the COVID-19 pandemic. Here, we demonstrate progressive C>U substitutions in SARS-CoV-2 genome within the timeframe of 5 months. We highlight the role of C>U substitutions in the reduction of 5′-UCG-3′ motifs and hypothesize that this progressive decrease is driven by host APOBEC activity.

We aligned 22164 SARS-CoV-2 genomes from GISAID database to the reference genome and observed a total of 9210 single-nucleotide changes with C>U being the most abundant (Figure 1A;Supplementary Text, Figures S1 and S2, and Table S1). Over a period of 5 months, we found a steady and substantial increase in C>U substitutions (Figure 1B), with almost half of them being synonymous (Supplementary Text and Figure S3), but not in other changes (Supplementary Figure S4). One potential driver behind the increase in C>U changes could be the recently proposed APOBEC-mediated viral RNA editing (Di Giorgio et al., 2020; Simmonds, 2020; Supplementary Text). Since APOBEC3 family members display a preference for RNA in open conformation as opposed to forming secondary structures (McDaniel et al., 2020), we calculated the folding potential of all genomic sites that include C>U substitutions (Figure 1C). Positions with C>U changes are more often located in regions with low potential for forming secondary RNA structures. These observations are in agreement with the notion that members of the APOBEC family are the main drivers of cytosine deamination in SARS-CoV-2 (Di Giorgio et al., 2020; Simmonds, 2020).

**Figure 1:**
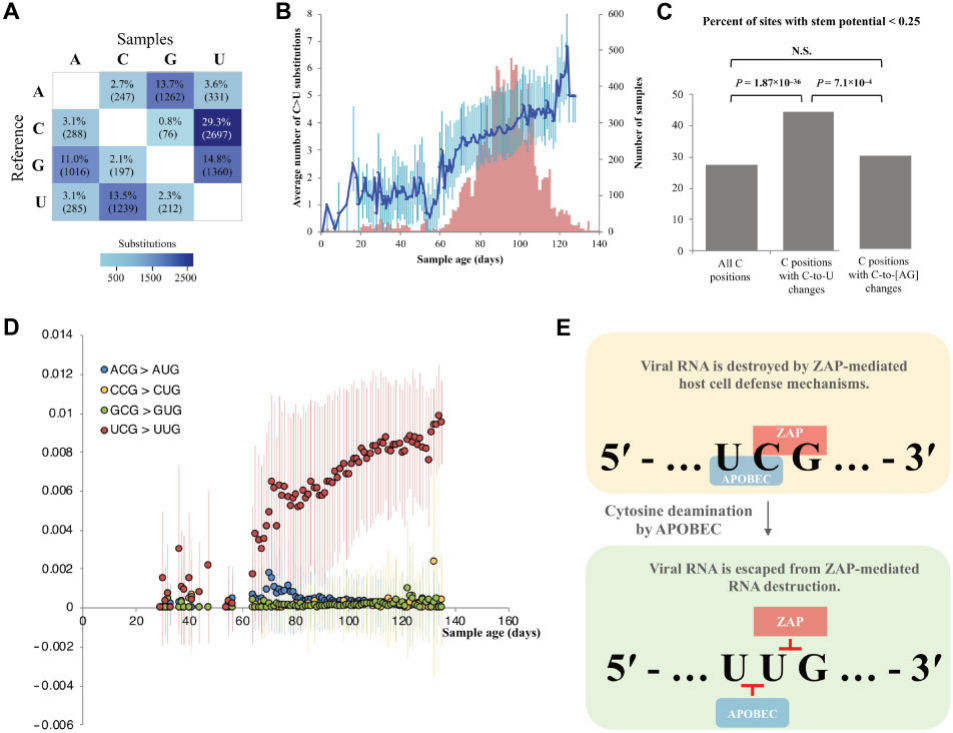
APOBEC-driven C>U substitutions in the SARS-CoV-2 genome contribute to CpG loss allowing viral RNA to escape from ZAP-mediated viral RNA destruction. (**A**) Single nucleotide variation events observed between individual SARS-CoV-2 sample sequences (*n *=* *22164) and the reference genome. (**B**) The number of C>U substitutions across sample dates. The average number of substitutions for each sampling day is plotted (blue line, left y-axis) with ±standard deviation (SD) as error bars. The number of samples for each day is shown as red bars (right y-axis). (**C**) Folding potential of positions with C>U changes (Supplementary Text). *P*-values from Fisher’s exact test are shown above bars. N.S., non-significant. (**D**) The fraction of [A/C/G/U]CG triplets that are changed to [A/C/G/U]UG over time. The average fractions, relative to the reference genome, are shown as circles for each sampling day (x-axis). Error bars denote ±SD. Only dates with at least 20 samples are plotted. (**E**) A model for the consequences of host-driven evolution by APOBEC enzymes on viral CpG dinucleotide composition.

We searched for possible APOBEC genetic footprints (5′-UC-3′>5′-UU-3′) in viral dinucleotide frequencies (Supplementary Figure S5). Among all dinucleotides, UpC showed the highest degree of decrease, while UpU exerted the highest rates of increase, which is consistent with APOBEC activity (Supplementary Text).

When analyzing the context of genomic sites undergoing C>U changes, we noticed an enrichment for 5′-UCG-3′ motifs (Supplementary Table S2). To assess the contribution of C>U changes in CpG loss, we examined the dynamics of [A/C/G/U]CG trinucleotides over time (Figure 1D). The progressive change (∼1% over a 5-month period) of 5′-UCG-3′ to 5′-UUG-3′ is most striking when supported by a larger number of genomes (Days 70‒115), whereas no such pattern is observed for the other trinucleotides (Figure 1D). The association between cytosine deamination and CpG loss is further underlined by the rapid, progressive increase in 5′-UCG-3′>5′-UUG-3′ changes compared to other 5′-UC[A/C/U]-3′ motifs (Supplementary Figure S6). The genomic region for the highest percentage of 5′-UCG-3′ loss is located in ORF1 (Supplementary Text and Figure S7). No apparent progression of 5′-UCG-3′ over time is observed on the negative strand, suggesting that the action of APOBEC on the negative strand of SARS-CoV-2 is limited compared to that on the positive strand (Supplementary Figure S8).

The zinc-finger antiviral protein (ZAP) selectively binds to viral CpG regions, resulting in viral RNA degradation (Takata et al., 2017). Previous studies reported that the reduced number of CpG motifs in HIV and other viruses played an important role in the viral replication inside the host cell, allowing the virus to escape ZAP protein activity (Takata et al., 2017). Similarly, a stronger suppression of CpGs is observed in SARS-CoV-2 compared to other coronaviruses (Digard et al., 2020). Given the high expression levels of APOBEC and ZAP genes in COVID-19 patients (Blanco-Melo et al., 2020), the direct interaction of APOBEC with viral RNA (Schmidt et al., 2021), and our observations, we hypothesize that as a consequence of APOBEC-mediated RNA editing, SARS-CoV-2 genome may escape host cell ZAP activity. Both APOBEC and ZAP are interferon-induced genes that act preferentially on ssRNA in open conformation (Luo et al., 2020; McDaniel et al., 2020). Initially, APOBEC and ZAP enzymes may have overlapping preferred target motifs for their enzymatic functions (Figure 1E). The catalytic activity of APOBEC on 5′-UC-3′ leads to cytosine deamination, which destroys ZAP’s specific acting site (5′-CG-3′). The conversion of C>U allows viral RNA to escape from ZAP-mediated RNA destruction. Therefore, uracil editing is more likely to become fixed at UCG positions due to the selective advantage this conveys to subvert ZAP-mediated degradation.

A recent study hypothesized that both ZAP and APOBEC provide selective pressure that drives the adaptation of SARS-CoV-2 to its host (Wei et al., 2020). Here, we provided one of the potential mechanisms that contribute to CpG reduction in SARS-CoV-2.

In summary, our phylogeny-free approach, together with other recent studies, strongly supports the proposed model and merits future experimental validation. To our knowledge, this is the first study linking the dynamics of viral genome mutation to two known host molecular defense mechanisms, the APOBEC and ZAP proteins.

*[Supplementary material is available at Journal of Molecular Cell Biology online. The data underlying this work are available in GISAID, at* *https://gisaid.org. The ID numbers of genomes used are provided in Supplementary Table S1. We thank all laboratories that have contributed sequences to the GISAID database and Zhadyra Yerkesh for giving her comments and helpful discussions. This work was supported by funding from King Abdullah University of Science and Technology (KAUST) R3T initiative. Work in A.P.’s laboratory is supported by the KAUST Faculty Baseline Fund (BAS/1/1020-01-01). A.P. supervised the project. M.S. and T.M. designed experiments. T.M. and Q.G. performed bioinformatic analysis. M.S. wrote the draft of the manuscript. All authors discussed, edited, read, and agreed to the final version of the manuscript.]*

## Supplementary Material

mjab011_Supplementary_Data
